# Supplementary material for: Reconciling Mining with the Conservation of Cave Biodiversity: A Quantitative Baseline to Help Establish Conservation Priorities
Source: PLoS One. 2016 Dec 20;11(12):e0168348. doi: 10.1371/journal.pone.0168348 (PMC5173368; doi:10.1371/journal.pone.0168348)
Supplement: S1 Dataset — (ZIP) [file pone.0168348.s002.zip › Taxa/Serra Norte/SN_2013/N5S/Lista N5S-61.pdf]

CAVIDADE N5S-0061

Classe	Ordem	Fam/Outros	Gên/Outros	Espécie	S-U	U-S	Zon.
Arachnida	Amblypygi	Phrynidae	*Heterophrynus*	*longicornis*	1		P
Arachnida	Araneae	Drymusidae		jovem		X	P
Arachnida	Araneae	Mysmenidae	*Microdipoena*	sp.1	X		P
Arachnida	Araneae	Ochyroceratidae	*Ochyrocera*	sp.1		X	P
Arachnida	Araneae	Salticidae		jovem	X		E
Arachnida	Araneae	Theridiosomatidae	*Plato*	sp.1	X	X	E/P
Diplopoda	Polydesmida	Pyrgodesmidae		jovem	X		P
Entognatha	Collembola	Paronellidae		sp.1	X		P
Entognatha	Collembola	Paronellidae		sp.4		X	P
Insecta	Diptera	Cecidomyiidae	Cecidomyiinae	sp.		X	E
Insecta	Diptera	Ceratopogonidae		sp.	X		P
Insecta	Diptera	Chironomidae		sp.	X		P
Insecta	Diptera	Dolichopodidae		sp.	X		E/P
Insecta	Diptera	Psychodidae	Phlebotominae	sp.		X	P
Insecta	Diptera	Sciaridae		sp.	X		
Insecta	Hymenoptera	Diapriidae		sp.1	X		P
Insecta	Hymenoptera	Formicidae	*Pachycondyla*	sp.2		X	E
Insecta	Hymenoptera	Formicidae	*Trachymyrmex*	sp.1	X		P
Insecta	Lepidoptera	Noctuoidea		sp.6		1	E
Insecta	Lepidoptera	Noctuoidea		sp.8	1		
Insecta	Orthoptera	Phalangopsidae	*Paraclodes*	sp.	1	3	E
Insecta	Orthoptera	Phalangopsidae	*Phalangopsis*	sp.		3	P
Amphibia	Anura			sp.	1		E
Mammalia	Chiroptera	Emballonuridae	*Peropteryx*	*kappleri*		1	E
